# Supplementary material for: Vaccines Against Urban Epidemic Arboviruses: The State of the Art
Source: Viruses. 2025 Mar 6;17(3):382. doi: 10.3390/v17030382 (PMC11945797; doi:10.3390/v17030382)
Supplement: Supplementary file 1 [file viruses-17-00382-s001.zip › viruses-3481575-supplementary.pdf]

Vaccines Against Urban Epidemic Arboviruses: The State of the Art

Cláudio Antônio de Moura Pereira[1], Poliana Gomes da Silva[1], Renata Pessôa Germano Mendes[1], Elton José Ferreira Chaves[1], Lindomar José Pena[1]

[1]Laboratory of Virology and Experimental Therapy (Lavite), Department of Virology, Aggeu Magalhães Institute (IAM), Oswaldo Cruz Foundation (Fiocruz), 50670-420, Recife, Pernambuco, Brazil.

*Corresponding author:

Lindomar Pena, DVM, PhD. Email: lindomarfiocruz@gmail.com or
lindomar.pena@fiocruz.br

Supplementary Material

VectorMap, a product of the Walter Reed Biosystematics Unit (WRBU), is the world's largest web-based repository for arthropod vector collection data, primarily focused on Aedes albopictus and Aedes aegypti [1]. This dataset includes significant recent surveillance data and combines literature-extracted information with specimen records from the United States National Museum (USNM) and WRBU collaborators [2].

Data description. Occurrence data for *Aedes aegypti* and *Aedes albopictus* were downloaded from VectorMap (vectormap.si.edu). Data were compiled from three sources: (i) museum specimen records, (ii) peer-reviewed literature, and (iii) publicly available mosquito surveillance records. VectorMap documents 93 fields of information for each record, including taxonomy, location site description, and collection method. A search of the VectorMap mosquito map service was carried out on July 16th, 2023. The use of Species = "aegypti" returned 33,734 total records, while Species = "albopictus" returned 44,831 total records.

Data preparation. The raw data was processed by means of a Python script using the "pandas" library. Specifically, three columns named "Species", "Decimal latitude" and "Decimal longitude" were selected and filtered by species type (*Aedes aegypti* or *Aedes albopictus*) to generate occurrence data for each species. Two additional data preparation procedures were then applied: (i) the elimination of duplicate entries (specimens captured at identical geographical coordinates); (ii) the geospatial coordinates of all data points were superimposed on a map outlining the administrative boundaries of the countries, including ocean regions. In addition, we removed any data points located over the ocean. Finally, the final dataset on the occurrence of *Aedes aegypti* (n = 9,923) and *Aedes albopictus* (n = 13,350) species did not contain duplicate records or georeferenced latitudes and longitudes that did not correspond to terrestrial land masses.

References

[1] Foley DH, Wilkerson RC, Birney I, Harrison S, Christensen J, Rueda LM. MosquitoMap and the Mal-area calculator: new web tools to relate mosquito species distribution with vector borne disease. International Journal of Health Geographics. 2010;9(1):11.

[2] Kraemer MUG, Sinka ME, Duda KA, Mylne A, Shearer FM, Brady OJ, Messina JP, Barker CM, Moore CG, Carvalho RG, et al. The global compendium of Aedes aegypti and Ae. albopictus occurrence. Scientific Data. 2015;2(1):150035.

[3] WHO/UNICEF Joint Monitoring Programme (JMP) for Water Supply and Sanitation – processed by Our World in Data. “Safely managed” [dataset]. WHO/UNICEF Joint Monitoring Programme (JMP) for Water Supply and Sanitation [original data].
